# Glucose Promotes EMMPRIN/CD147 and the Secretion of Pro-Angiogenic Factors in a Co-Culture System of Endothelial Cells and Monocytes

**DOI:** 10.3390/biomedicines12040706

**Published:** 2024-03-22

**Authors:** Fransis Ghandour, Sameer Kassem, Elina Simanovich, Michal A. Rahat

**Affiliations:** 1 Department of Internal Medicine A, Carmel Medical Center, Haifa 3436212, Israel; 2 The Rappaport Faculty of Medicine, Technion-Israel Institute of Technology, Haifa 3109601, Israel; 3 Immunotherapy Laboratory, Carmel Medical Center, Haifa 3436212, Israel

**Keywords:** diabetes mellitus, CD147/EMMPRIN, endothelial cells, glucose, metformin, AMPK, dorsomorphin/compound C

## Abstract

Vascular complications in Type 2 diabetes mellitus (T2DM) patients increase morbidity and mortality. In T2DM, angiogenesis is impaired and can be enhanced or reduced in different tissues (“angiogenic paradox”). The present study aimed to delineate differences between macrovascular and microvascular endothelial cells that might explain this paradox. In a monoculture system of human macrovascular (EaHy926) or microvascular (HMEC-1) endothelial cell lines and a monocytic cell line (U937), high glucose concentrations (25 mmole/L) increased the secretion of the pro-angiogenic factors CD147/EMMPRIN, VEGF, and MMP-9 from both endothelial cells, but not from monocytes. Co-cultures of EaHy926/HMEC-1 with U937 enhanced EMMPRIN and MMP-9 secretion, even in low glucose concentrations (5.5 mmole/L), while in high glucose HMEC-1 co-cultures enhanced all three factors. EMMPRIN mediated these effects, as the addition of anti-EMMPRIN antibody decreased VEGF and MMP-9 secretion, and inhibited the angiogenic potential assessed through the wound assay. Thus, the minor differences between the macrovascular and microvascular endothelial cells cannot explain the angiogenic paradox. Metformin, a widely used drug for the treatment of T2DM, inhibited EMMPRIN, VEGF, and MMP-9 secretion in high glucose concentration, and the AMPK inhibitor dorsomorphin enhanced it. Thus, AMPK regulates EMMPRIN, a key factor in diabetic angiogenesis, suggesting that targeting EMMPRIN may help in the treatment of diabetic vascular complications.

## 1. Introduction

Type 2 diabetes mellitus (T2DM) is a global pandemic that is associated with enormous social and economic burden [1]. The worldwide prevalence of diabetes was estimated to be 529 million in 2021 and is increasing [2]. Morbidity and mortality in diabetic patients are mostly attributed to vascular complications, and the cardiovascular risk of subjects with diabetes is estimated to be increased 2–4-fold compared to non-diabetics [3]. The hallmark of T2DM is chronic hyperglycemia, which is associated with metabolic, inflammatory, and tumorigenic processes [4]. Chronic hyperglycemia and metabolic changes activate molecular pathways, including the activation of the protein kinase C (PKC), polyol, and hexosamine pathways, the increased production of reactive oxygen species (ROS), and the accumulation of advanced glycation end products (AGEs) that lead to persistent inflammation [5]. The response of endothelial cells to hyperglycemia causes their dysfunction and ultimately their death of glucose toxicity [5,6]. In small blood vessels, this causes damage that results in retinopathy, nephropathy, and neuropathy. In large blood vessels, macrovascular damage increase cerebrovascular, coronary, and peripheral vascular diseases [5]. Since endothelial cells line the inner lumen of blood vessels, they are at the forefront of metabolic and inflammatory derangements of diabetes, especially hyperglycemia-induced glucotoxicity, which includes non-enzymatic glycation, oxidative stress, leukocyte migration, and enhanced inflammation [7]. 

Angiogenesis is a complex process of the budding of new blood vessels from existing ones, and is necessary to repair wounds and damage to blood vessels. However, in patients with diabetes, angiogenesis is impaired, and can be either excessive or insufficient in different tissues, leading to different pathologies [8]. Endothelial dysfunction has been described as an early sign of vascular disease [9], and in diabetic patients, hyperglycemia was shown to enhance endothelial dysfunction [8,10,11,12], and lead to overall reduction in angiogenesis in many tissues and to a reduced ability to heal wounds or to grow coronary collateral vessel during ischemic events. These responses lead to an increased risk for cardiovascular events, the defective formation of granulation tissue during wound healing, and limb amputations [13]. On the other hand, angiogenesis is enhanced during diabetic retinopathy and early stages of diabetic nephropathy [14]. This tissue-specific regulation of angiogenesis has been termed ‘the diabetic angiogenic paradox’ [15,16]. Some differences in the hyperglycemia-triggered signaling pathways between microvascular and macrovascular endothelial cells might explain this paradox; however, the reasons for the differences between the angiogenic responses of macrovascular and microvascular endothelial cells still remain mostly unclear, and may depend on the local microenvironment and interactions with other cell types. 

Metformin is used as a first-line drug for the treatment of T2DM worldwide. It has multiple mechanisms of action including its ability to reduce hyperglycemia and hyperlipidemia, inhibit liver glycogenesis, and increase the uptake of glucose in the peripheral tissues [17]. Additionally, metformin also has anti-cancerous and anti-inflammatory effects [18]. Since angiogenesis is strongly linked to inflammation and hypoxia, and many inflammatory genes also have a pro-angiogenic activity, it was suggested that metformin also affects angiogenesis [19]. Some evidence suggests that metformin can promote angiogenesis by increasing vascular endothelial growth factor (VEGF) expression and signaling, but it can also inhibit angiogenesis by inhibiting endothelial cell proliferation and migration, and the secretion of matrix metalloproteinases (MMPs) [20,21]. These effects are mediated, at least partially, by the activation of AMP-activated protein kinase (AMPK) [22,23]. The actual effect of metformin on angiogenesis might depend on the organ, the cell type, the dosage, or the microenvironment. Those effects, specifically in the context of hyperglycemia, are not yet sufficiently studied. 

Extracellular matrix metalloproteinase inducer (EMMPRIN, or CD147) is a transmembrane glycoprotein that is expressed on the surface of many cell types, including endothelial cells. EMMPRIN participates in many cellular processes. Especially relevant is its ability to promote aerobic glycolysis in cells, as it chaperones the monocarboxylate transporters MCT1/4 to the plasma membrane [22]. Glucose uptake that is controlled by the level of expression of the glucose transporter GLUT1 may be enhanced by EMMPRIN that inhibits the p53-mediated degradation of Glut1 [23], or inhibited by the inhibitory effects of EMMPRIN on Glut1 expression [24]. EMMPRIN can also induce the expression of MMPs, which degrade the extracellular matrix, enable the migration of endothelial cells, a key step in angiogenesis, and promote the production of the potent pro-angiogenic factor VEGF [25]. The various functions of EMMPRIN depend on its glycosylation on three N-linked glycosylation sites [26]. EMMPRIN expression is increased in various types of cancer, which are often associated with increased angiogenesis, and it has been implicated in the pathogenesis of other diseases that involve abnormal angiogenesis, such as diabetic retinopathy and psoriatic arthritis [27,28]. However, the angiogenic role EMMPRIN plays in diabetes is not well understood. 

In this study, we aimed to explore the effect of hyperglycemia on the secretion of pro-angiogenic factors from micro- versus macro-vascular endothelial cells using an in vitro model, in the context of inflammation induced by co-culturing with monocytes. Understanding the mechanisms underlying angiogenesis in diabetes may lead to new therapies for preventing or treating diabetic complications.

## 2. Materials and Methods

Cells: The human EaHy926 macrovascular endothelial cell line (ATCC CRL-2922) and the human HMEC-1 microvascular endothelial cell line (ATCC CRL-3243) were cultured alone and in a co-culture with the human monocyte-like U937 cells (ATCC 1593). Both endothelial cell lines were grown in high-glucose Dulbecco’s Modified Eagle’s Medium (DMEM), with 10% fetal calf serum (FCS), 1% amphotericin B, 1% L-glutamine, 2% HAT supplement (a mixture of hypoxanthine, aminopterin, and thymidine), and 1% antibiotics (Penicillin-Streptomycin-Neomycin). The U937 cells were cultured in RPMI-1640 medium, 10% FCS, 1% amphotericin B, and 1% antibiotics. All tissue culture reagents were purchased from Biological Industries, Beit Ha’emek, Israel. The cells were split twice a week at a ratio of 1:4, and were routinely checked for the presence of mycoplasma. Cell viability was tested using the XTT kit (Biological Industries).

Before co-culturing, endothelial cells were extensively washed, trypsinized, and then seeded in 96-well or 24-well plates (20,000 or 100,000 cells/well, respectively) overnight and allowed to adhere. Then, the medium was replaced with low-glucose DMEM with 1% Glutamine and without the addition of FCS (serum starvation medium), and an equal number of U937 cells was added to this medium, with glucose supplemented to the final concentration as indicated. In some experiments, metformin hydrochloride (2 mmole/L, Teva, Tel-Aviv, Israel), the rabbit anti-human EMMPRIN (h161-pAb, 2 ng/mL) that was produced in our lab, or the AMPK inhibitor dorsomorphin (compound C, Merck KGaA, Darmstadt, Germany) were added to some of the wells, as indicated. The h161-pAb was purified on a protein A column from the serum of a rabbit immunized with a peptide derived from the human EMMPRIN sequence (amino acids 52–62), as previously described [29]. After an incubation of 24 h, supernatants were collected for the determination of EMMPRIN, VEGF, and MMP-9 concentrations. The viability of the cells was determined using the XTT kit (Biological Industries). 

ELISA: Concentrations of EMMPRIN, VEGF, and MMP-9 were measured using the commercial DuoSet ELISA kits (R&D systems, Minneapolis, MN, USA), according to the manufacturer’s instructions. Supernatant samples in duplicates were diluted 1:100, as determined in preliminary calibration experiments. The phosphorylation of AMPK was tested using the DuoSet intracellular human phopsho-AMPKα1 kit (R&D systems), according to the manufacture’s protocol. 

Wound Assay: EaHy926 cells were seeded (2 × 10^4^ cells/well) in 96-well plates and cultured to confluency. The monolayer was then scratched using a toothpick, and the non-adherent cells were washed away with PBS. Then, the supernatants obtained from EaHy926 cells treated with different concentrations of glucose, with or without the anti-EMMRIN antibody (2 ng/mL), were diluted (1:2) and added to the scratched endothelial layer. Images of the scratch site were acquired immediately after scratching the monolayer (T0) and 18 h later (T18) (Moticam 2MP, magnification ×4), and the length to which the cells migrated was measured using the ImagePro plus 4.5 software (Media Cybernetics, Inc., Rockville, MD, USA). 

Statistical analyses: All values are presented as means ± standard error of mean (SEM). Significance between groups was determined by the one-way analysis of variance (ANOVA) test, followed by Bonferroni’s multiple post hoc comparison test, using Prism 9.3 (GraphPad software, Boston, MA, USA). *p* values exceeding 0.05 were not considered significant. 

## 3. Results

### 3.1. Glucose Enhances Endothelial Cell Death at Very High Concentrations 

Hyperglycemia in T2DM patients could affect the function of both monocytes and endothelial cells. Indeed, the pro-inflammatory activation of monocytes and enhanced vascular inflammation has been reported in T2DM patients [30,31]. Thus, we opted to use a co-culture system of monocytes and endothelial cells to examine the effects of high glucose levels on their interactions. 

Chronic hyperglycemia may lead to the glucose toxicity of cells [5,6], and therefore, we first determined the range of glucose concentrations that could be used in this study without leading to cell death. We incubated single cell cultures in physiologic-glucose DMEM that contains 5.5 mmole/L glucose (equivalent to 90 mg/dL), or with increased glucose concentrations. 

**Figure 1:**
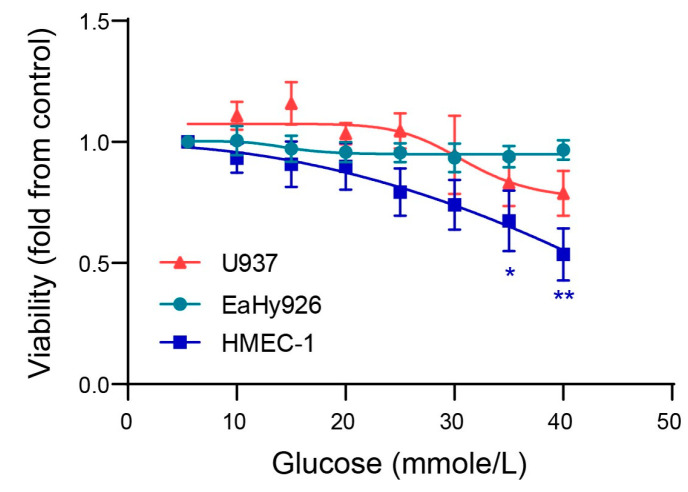
Glucose affects only microvascular endothelial cell viability at high concentrations. Cells of the three cell lines (20,000 cells/well) were seeded separately in low-glucose DMEM, with increasing concentrations of glucose, and after 24 h of incubation, cell viability was determined using the XTT assay. Results are presented as fold from control of each cell line. The one-way ANOVA followed by Dunnett’s multiple comparisons test was used (*n* = 7 for each cell line at each time point). *, *p* < 0.05, **, *p* < 0.01 relative to no treatment.

Glucose had no significant effect on EaHy926 and U937 cell viability even at high concentrations, whereas cell death in the microvascular HMEC-1 endothelial cells significantly occurred at concentrations higher than 30 mmole/L (equivalent to 540 mg/dL) (Figure 1). Therefore, in our subsequent experiments, we limited our use of glucose concentrations to three concentrations, representing normal glycemia (5.5 mmole/L or 99 mg/dL), moderately uncontrolled diabetes (10 mmole/L or 180 mg/dL), and the severely uncontrolled hyperglycemic state (25 mmole/L or 450 mg/dL).

### 3.2. High Glucose Concentrations Enhance the Secretion of Pro-Angiogenic Factors from Endothelial Cells

**Figure 2:**
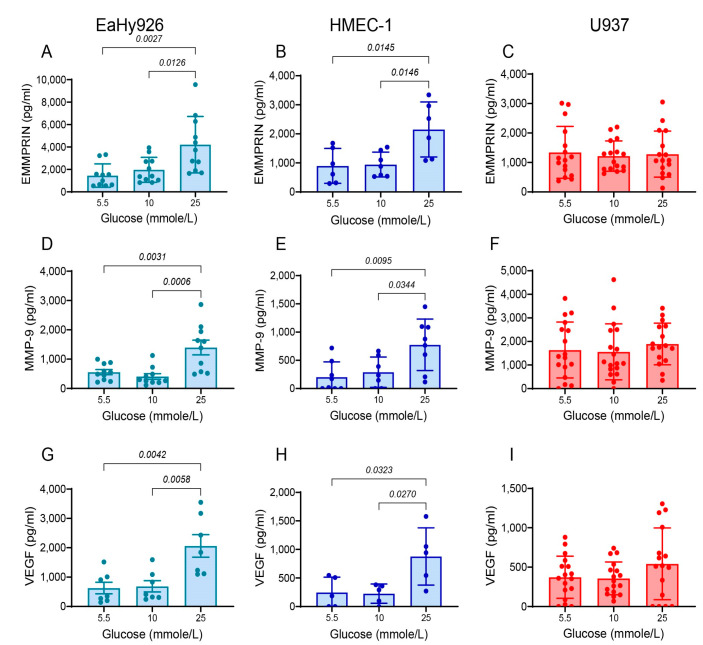
High glucose concentrations enhance the expression of EMMPRIN, MMP-9, and VEGF in endothelial cells. The human macrovascular endothelial cells EaHy926, microvascular endothelial cells HMEC-1, and the monocytic-like cell line U937 were incubated as single cultures (100,000 cells each/well/500 μL) in serum-starvation medium with low-glucose DMEM, and glucose was added to achieve the indicated concentrations. Cells were incubated for 24 h, and then superannuates were collected and the concentrations of (**A**–**C**) EMMPRIN, (**D**–**F**) MMP-9, and (**G**–**I**) VEGF, were determined using ELISA. The means ± SEM are presented (*n* = 7). Data were analyzed using the one-way ANOVA followed by Bonferroni’s post hoc test.

We next explored the effect of glucose concentrations on the secretion of the pro-angiogenic factors EMMPRIN, MMP-9, and VEGF, in each of the cell lines. Monocultures of both the macrovascular endothelial cell line EaHy926 (Figure 2A,D,G) and the microvascular endothelial cell line HMEC-1 (Figure 2B,E,H) demonstrated no difference between the levels of these factors when incubated with 5.5 mmole/L versus 10 mmole/L glucose. However, a significant increase in the secreted levels of EMMPRIN, MMP-9, and VEGF was observed in both endothelial cell lines upon incubation with 25 mmole/L glucose. In contrast, the level of the three pro-angiogenic factors remained constant in the monocytic-like cell line U937 (Figure 2C,F,I), regardless of the change in glucose concentrations. Thus, endothelial cells are more sensitive to high glucose concentrations than monocytes, and respond by enhancing the secretion of pro-angiogenic factors. 

### 3.3. Co-Culturing of Endothelial Cells and Monocytes Enhances Secretion of Pro-Angiogenic Factors

**Figure 3:**
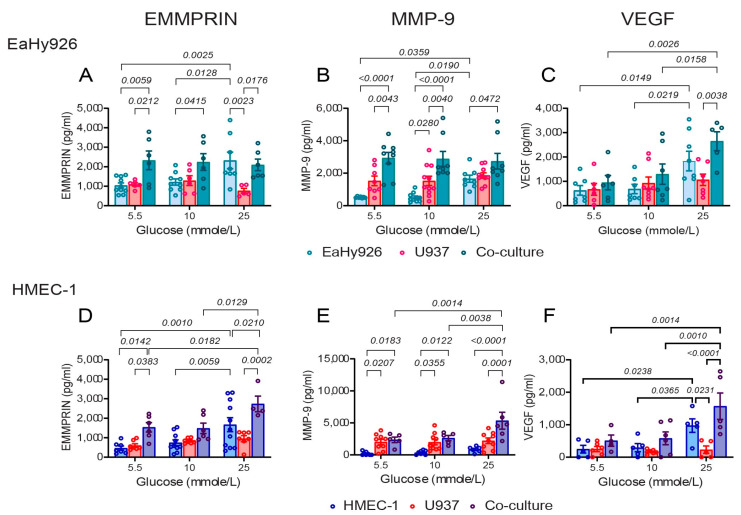
High glucose concentrations and co-culturing of endothelial cells with monocytes enhance the secretion of EMMPRIN, MMP-9, and VEGF. The human endothelial cells EaHy926 and HMEC-1, derived from macrovasculature or microvasculature, respectively, were incubated for 24 h alone (100,000 cells each/500 μL in a 24-well plate) or in a co-culture with the human monocytic-like cell line U937 (100,000 cells) in low-glucose DMEM serum-starvation medium, with increasing glucose added to achieve the indicated concentrations. At the end of the incubation, superannuates were collected and the concentrations of (**A**,**D**) EMMPRIN, (**B**,**E**) MMP-9, and (**C**,**F**) VEGF, were determined using ELISA. The means ± SEM are presented (*n* = 6). Data were analyzed using the one-way ANOVA followed by Bonferroni’s post hoc test.

Since endothelial cells interact with monocytes within the blood vessel, we next asked whether monocytes could induce the endothelial cells to secrete more EMMPRIN, MMP-9, and VEGF upon co-culturing in the presence of the three glucose concentrations. With the EaHy926 cells, co-culturing with the U937 monocytes showed the same EMMPRIN and MMP-9 levels for all glucose levels, and their secreted levels at 5.5 mmole/L glucose was not different from those observed for the single EaHy926 culture at 25 mmole/L glucose (Figure 3A,B). The secretion of VEGF did not change in single or co-cultures at low glucose concentrations, and was enhanced at 25 mmole/L (Figure 3C). The co-incubation of the microvascular cell line HMEC-1 with the U937 in the two lower glucose concentration levels (5.5 and 10 mmole/L) showed the same results as in the EaHy926 cells. But the higher glucose level (25 mmole/L) enhanced the secretion of EMMPRIN and MMP-9 above the levels observed in this glucose concentration when HMEC-1 cells were incubated alone, and enhanced the secretion of VEGF compared to the co-culture in lower glucose levels (Figure 3D–F). Thus, VEGF is influenced by high glucose levels, whereas EMMPRIN and MMP-9 are influenced by either glucose or co-culture. An additive effect between high glucose levels and co-culture was observed only in the microvascular HMEC-1 for EMMPRIN and MMP-9. 

Since both endothelial cell lines secreted the highest levels of the pro-angiogenic factors when co-cultured with the monocytes, we used the co-culture system in the following experiments.

### 3.4. EMMPRIN Mediates the Effects of High Glucose Concentration and of the Co-Culture on the Secretion of Pro-Angiogenic Factors and Endothelial Cell Migration

**Figure 4:**
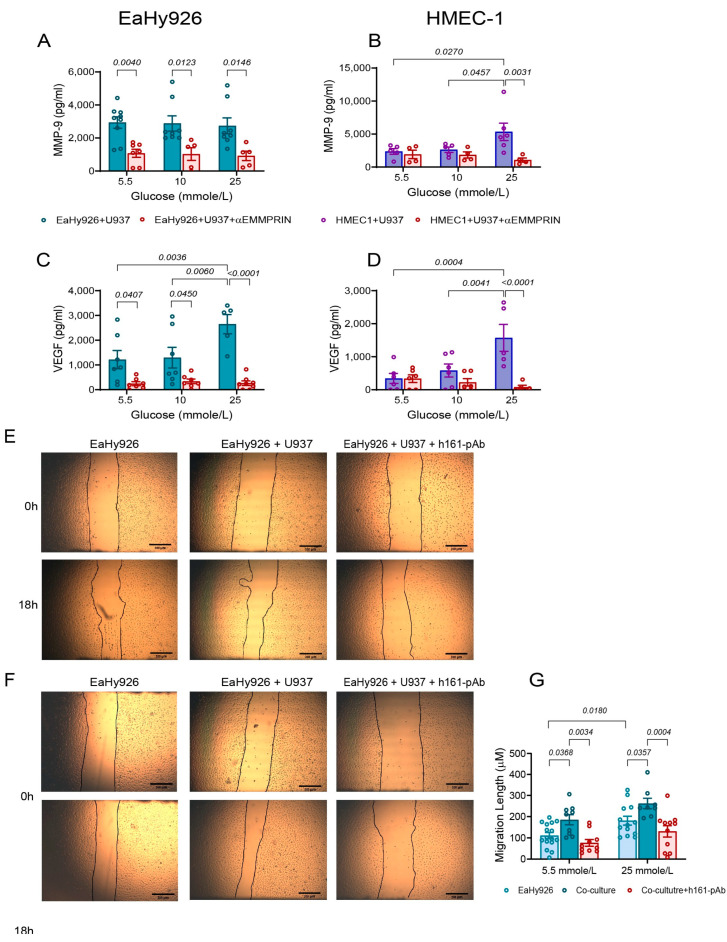
The anti-EMMPRIN antibody (h161-pAb) decreases VEGF and MMP-9 and cell migration. (**A**–**D**) The human endothelial cells EaHy926 or HMEC-1 (100,000 cells/well) were incubated in co-culture with the U937 cells at a 1:1 ratio, with glucose added to achieve the indicated concentrations, and with or without the addition of the anti-EMMPRIN antibody (h161-pAb, 2 ng/mL). Cells were incubated for 24 h, and the concentrations of (**A**,**B**) MMP-9 and (**C**,**D**) VEGF were determined using ELISA. The means ± SEM are presented (*n* = 4–7). Data were analyzed using the one-way ANOVA followed by Bonferroni’s post hoc test. (**E**–**G**) The human endothelial cell line EaHy926 (20,000 cells/well) was seeded and allowed to grow to confluency overnight. A scratch was made with a toothpick, and non-adherent cells were washed away. The cells were then incubated for 18 h with full medium and supernatants (diluted at a ratio of 2.5:1) derived from EaHy926 cells that were previously incubated alone or in co-culture with glucose and with or without the h161-pAb (2 ng/mL). Representative images of (**E**) assay at 5 mmole/L glucose, (**F**) assay at 25 mmole/L glucose, and (**G**) quantitative analysis of the assays (*n* = 8–12). The distance to which cells migrated was calculated from images taken at 0 h and 18 h. The means ± SEM are presented, and data were analyzed using the one-way ANOVA followed by Bonferroni’s post hoc test.

EMMPRIN is known to induce both MMP-9 and VEGF, and therefore we asked whether EMMPRIN mediates the effects of glucose on the co-cultured endothelial cells. To this end, we co-cultured the endothelial cells with monocytes in the presence of different glucose concentrations, and added the neutralizing anti-EMMPRIN antibody (h161-pAb), which we had previously developed in our lab. As seen before, co-cultured the EaHy926 cells exhibited high levels of secreted MMP-9 regardless of the glucose levels, whereas VEGF secretion was elevated only in high glucose levels (25 mmole/L). The h161-pAb antibody inhibited the secretion of MMP-9 and VEGF in all glucose concentrations (Figure 4A,C). In the HMEC-1 cells, the increased levels of MMP-9 and VEGF at co-cultures incubated at glucose concentrations of 5.5 and 10 mmole/L were further increased at the 25 mmole/L concentration (as seen in Figure 3D–F), and the anti-EMMPRIN antibody reduced these levels only at the 25 mmole/L glucose concentration (Figure 4B,D). When the antibody was added to monocultures, the inhibitory effect on VEGF and MMP-9 secretion was observed only in the high glucose levels of both endothelial cells, but in the monocytes, only MMP-9 was inhibited by the antibody (Figure S1). 

To validate that this increase in the pro-angiogenic factors was also reflected in a functional assay, we used the wound healing assay to monitor changes in the proliferation/migration ability of the endothelial cells. A confluent monolayer of EaHy926 cells was scratched and then incubated with supernatants obtained from EaHy926 cells that were cultured alone or in co-culture in a medium containing 5.5 or 25 mmole/L glucose, with or without the addition of the anti-EMMPRIN antibody. After 18 h of incubation, the distance to which the wounded endothelial cell layer migrated was determined. We show that the angiogenic potential of the EaHy926 was increased by the co-culture, but even more so by the combined effect of the co-culture and the high glucose concentration of 25 mmole/L (Figure 4E–G). However, in both cases, the addition of the anti-EMMPRIN antibody inhibited this increased migration by about 2-fold, suggesting that EMMPRIN is involved in mediating the effects of the co-culture. Similar results were also obtained for the HMEC-1 cell lines (Figure S2). 

### 3.5. Metformin Inhibits the Pro-Angiogenic Effects of High Glucose Concentration or of the Co-Culture

**Figure 5:**
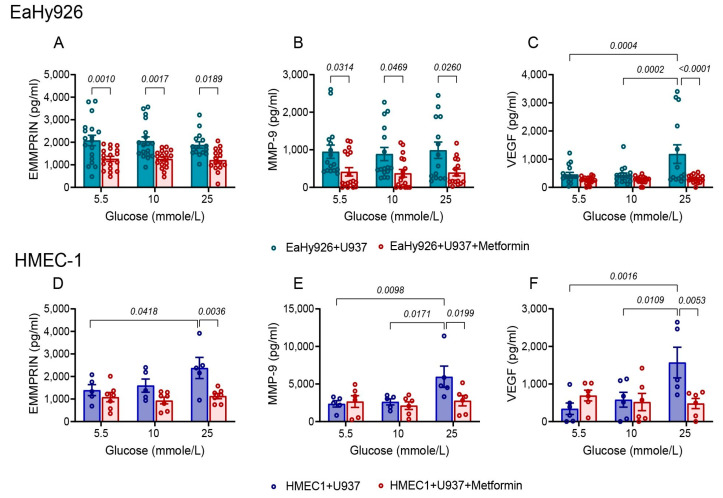

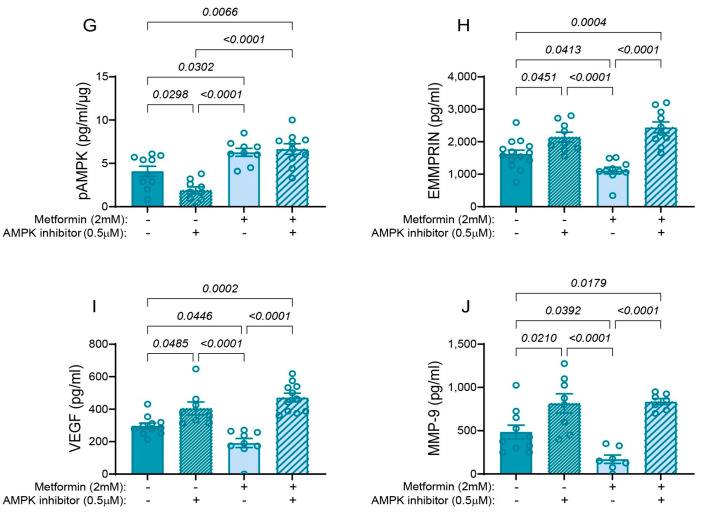
Metformin inhibits the secretion of the pro-angiogenic factors. (**A**–**F**) The human endothelial cells EaHy926 or HMEC-1 (100,000 cells/well/500 μL) were co-cultured with the U937 cells at a 1:1 ratio, with increasing glucose added to achieve the indicated concentrations, and with or without the addition of metformin (2 mmole/L). Cells were incubated for 24 h, and the concentrations of (**A**,**D**) EMMPRIN, (**B**,**E**) MMP-9, and (**C**,**F**) VEGF were determined using ELISA. The means ± SEM are presented (for EaHy926 cells, *n* = 10–15 in each group; for HMEC-1 cells, *n* = 5–6 in each group). Data were analyzed using the one-way ANOVA followed by Bonferroni’s post hoc test. (**G**–**J**) The human endothelial cells EaHy926 (100,000 cells/well/1000 μL) were co-cultured with the U937 cells at a 1:1 ratio in inserts (0.4 μm pore size) with 25 mmole/L glucose, and with or without the addition of metformin (2 mmole/L), the AMPK inhibitor (0.5 μM), or their combination. Cells were incubated for 24 h, and then supernatants were collected and cells were lysed. (**G**) The phosphorylation of AMPK was determined using ELISA (*n* = 8–12). The concentrations of (**H**) EMMPRIN, (**I**) VEGF, and (**J**) MMP-9 in the supernatants were determined using ELISA (*n* = 7–9 in each group). The means ± SEM are presented. Data were analyzed using the one-way ANOVA followed by Bonferroni’s post hoc test.

Metformin is a common drug administered to diabetic patients. However, its effects on angiogenesis are not fully understood. We used our co-culture system to learn about the effects of metformin on the secretion of EMMPRIN, MMP-9, and VEGF. We show that metformin inhibits the secretion of EMMPRIN and MMP-9 from EaHy926 cells at the three concentrations of glucose tested (Figure 5A,B), but VEGF secretion is affected only in the presence of high glucose concentrations (Figure 5C). In contrast, in the HMEC-1 cells, metformin inhibits the secretion of the three pro-angiogenic factors only at the high glucose concentration of 25 mmole/L (Figure 5D–F). 

### 3.6. Metformin Inhibits the Pro-Angiogenic Factors via Activation of AMPK

Since we showed that EMMPRIN mediates the interactions between endothelial cells and monocytes, and that metformin inhibits this effect at high glucose levels, we next asked about the molecular mechanism responsible. The main mode of action of metformin is its ability to activate the adenosine-monophosphate-activated protein kinase (AMPK) [32], but its effect on pro-angiogenic factors is less studied. To determine whether the inhibitory effect of metformin on EMMPRIN, VEGF, and MMP-9 secretion is mediated by the activation of AMPK, we incubated the macrovascular EaHy926 cells and U937 monocytes in medium with high glucose concentration (25 mmole/L) in inserts (0.4 μm pore size) that allowed the extraction of protein lysates from each cell type separately.

The phosphorylation of the Thr183 residue of the AMPKα1 subunit is necessary for the activation of the enzyme [33]. As expected, the AMPK inhibitor dorsomorphin inhibited the phosphorylation of the Thr183 residue of AMPKα1 subunit in EaHy926 cells, and the addition of metformin stimulated it (Figure 5G). However, the combination of the AMPK inhibitor and metformin resulted in the phosphorylation of AMPK, suggesting that metformin operates upstream of AMPK and regulates its phosphorylation. 

The AMPK inhibitor increased the secretion of EMMPRIN, VEGF, and MMP-9, while metformin inhibited it, relative to the untreated co-culture. Thus, AMPK activity inhibits the secretion of the pro-angiogenic factors. However, relative to the addition of the AMPK inhibitor alone, there was no difference in the secretion of the pro-angiogenic factors when AMPK inhibitor and metformin were combined (Figure 5H–J), again suggesting that metformin works upstream of AMPK. 

## 4. Discussion

In this study, we show in our in vitro co-culture system that high glucose levels, that are characteristic of untreated diabetic patients (25 mmole/L that are equivalent to 450 mg/dL), in the presence of inflammatory cells such as monocytes, can trigger endothelial cells to increase their secretion of pro-angiogenic factors and promote angiogenesis. This response could contribute to the development of diabetic complications, such as diabetic retinopathy or early nephropathy. We focus on EMMPRIN, a pro-angiogenic protein that can induce the expression and secretion of the two potent pro-angiogenic factors VEGF and MMP-9, and we demonstrate that the interactions between endothelial cells and monocytes are mediated by EMMPRIN. Furthermore, metformin, a common drug used to treat T2DM, promotes AMPK phosphorylation and activity, which in turn inhibits the secretion of EMMPRIN, VEGF, and MMP-9. Thus, metformin inhibits angiogenesis indirectly via AMPK and EMMPRIN in our system. 

The tissue-specific angiogenic paradox in T2DM describes the excessive formation of immature blood vessels in tissues such as the retina or kidney, and the simultaneous deficiency in small blood vessels in peripheral tissues, such as the skin, that lead to ischemia or impaired wound healing response [12]. However, the molecular basis for this paradox remains unclear. The minor differences we found between the HMEC-1 microvascular endothelial cells and EaHy926 macrovascular endothelial cells cannot account for this paradox, as both macrovascular and microvascular endothelial cells exhibited mostly similar trends. In fact, both EaHy926 and HMEC-1 demonstrated increased angiogenic potential when exposed to high glucose levels in the presence of monocytic cells (Figure 3 and Figure 4), suggesting that this in vitro co-culture system better simulates diabetic retinopathy than diabetic ischemia. 

Only minor differences between the macrovascular EaHy926 cells and the microvascular HMEC-1 cells were found. First, the microvascular HMEC-1 cells were more sensitive to the very high glucose concentrations (above 30 mmole/L) and underwent enhanced cell death (Figure 1). This is consistent with the glucotoxicity previously reported in brain microvascular endothelial cells [34,35]. In contrast, the macrovascular endothelial cells, as well as the monocytic cells, were resistant to the detrimental effects of such high glucose concentrations. The reasons for this difference in susceptibility to high glucose levels are not fully understood, but we speculate that one of the factors contributing to this difference may be a more efficient antioxidant defense system in the EaHy926 cells that better protects against oxidative stress induced by high glucose levels [35]. 

Secondly, the combination of the co-culture with high glucose levels resulted in an additive and maximal secretion of both EMMPRIN and MMP-9 in HMEC-1 cells, while maximal VEGF secretion required only the high glucose levels. In contrast, the secretion of EMMPRIN and MMP-9 from the co-cultured macrovascular EaHy926 cells was insensitive to glucose levels, and similar to their secretion in a single culture with high glucose levels, whereas VEGF secretion was dependent only on the incubation in high glucose concentrations (Figure 3). 

Thirdly, in EaHy926 co-cultures, the anti-EMMPRIN antibody (h161-pAb) inhibited the secretion of MMP-9 and VEGF in all glucose concentrations, whereas in the HMEC-1 co-cultures, h161-pAb was effective only at the 25 mmole/L glucose concentration (Figure 4). Metformin inhibited the secretion of EMMPRIN and MMP-9 from EaHy926 cells even at low concentrations of glucose, whereas in HMEC-1 cells, it was effective only at the high glucose concentration of 25 mmole/L (Figure 5). 

These minor differences between EaHy926 and HMEC-1 cells are not easily explained, and may be inherent to the properties of each specific cell line. However, it is clear that high glucose levels, the hallmark of T2DM, in the context of inflammation, simulated here through the interactions with monocytic cells, promote angiogenesis that may lead to diabetic complications. Accordingly, both the anti-EMMPRIN antibody and metformin are effective in inhibiting the secretion of the three pro-angiogenic factors in such pathological conditions. 

EMMPRIN is an adhesion molecule that mediates interactions between different cell types. We and others have previously demonstrated that EMMPRIN mediates the interactions between monocytes and tumor cells [36,37,38], and between monocytes and fibroblasts [28,39]. EMMPRIN can induce the expression and activity of the potent pro-angiogenic factor VEGF in tumor cells, fibroblasts, and monocytes by activating the PI3K/Akt pathway [40,41], and to increase the soluble isoforms of VEGF in endothelial cells [42]. Likewise, EMMPRIN stimulates MMP-9 expression in tumor cells, fibroblasts, and monocytes by activating the ERK and NF-κB pathways [25,43]. Here, we have demonstrated that EMMPRIN also mediates the interactions between endothelial cells and monocytic cells. 

The role of EMMPRIN in mediating interactions between endothelial cells and leukocytes, especially in the context of T2DM, was not investigated in depth. Enhanced EMMPRIN expression was identified in endothelial cells, infiltrating leukocytes and myofibroblasts in diabetic retinas [27], and the high-glycosylated EMMPRIN was shown to enhance the adhesion of monocytes to endothelial cells [44]. The enhanced expression of EMMPRIN in T2DM patients could also arise from the increased serum levels of insulin-like growth factor-1 (IGF-1) [45] or other factors yet unidentified. Interestingly, there is a positive feedback loop between EMMPRIN/CD147 and IGF-I [46], where EMMPRIN enhances IGF-1 expression and retinal angiogenesis [47], and IGF-1 increases EMMPRIN expression [48]. However, we did not explore the role of IGF-1 or other factors in this study. Moreover, the ability of the neutralizing anti-EMMPRIN antibody to inhibit VEGF and MMP-9 secretion and the angiogenic potential demonstrated here (Figure 4 and Figure 5), suggest that EMMPRIN also regulates these two pro-angiogenic factors in endothelial cells. Our assumption that EMMPRIN, VEGF, and MMP-9 contribute to the development of diabetic microvascular complications such as retinopathy is further supported by the evidence of elevated serum levels of EMMPRIN in diabetic patients [27,49]. Thus, EMMPRIN is a key factor, not only in cancerous diseases where it is mostly studied, but also in the context of metabolic and inflammatory diseases, such as T2DM. The ability of the anti-EMMPRIN antibody (h161-pAb) to inhibit VEGF and MMP-9 secretion (Figure 4) and to attenuate endothelial cell migration and proliferation, as observed in the wound assay (Figure 4 and Figure S2), suggests that it may hold a potential therapeutic value for the treatment of diabetic vascular complications.

Metformin is the most prescribed medication for T2DM, with a wide range of activities. The main mechanism of action of metformin is the activation of liver kinase B1 (LKB1) that can phosphorylate the Thr183 residue on the α1 subunit of AMPK, thus activating the enzyme [33]. This leads to the inhibition of gluconeogenesis and lipogenesis and the reduction of insulin resistance in muscle and fat tissues. Additionally, metformin can regulate inflammation, attenuate cell proliferation, and improve oxidative status [50]. Beyond its anti-diabetic properties, metformin can exert favorable effects on endothelial function [51] and improve cardiovascular risk [52]. Moreover, the use of metformin was associated with reduced cancer risk and improved survival in some cancers [53,54]. These effects of metformin may be partially mediated by its inhibitory effects on pro-angiogenic factors in the context of hyperglycemia, as demonstrated in our study. This is supported by previous findings that showed reduced MMP-9 and VEGF secretion after metformin treatment that was mediated by AMPK [55,56,57]. 

We show that in the context of hyperglycemia, metformin reduced the secretion of EMMPRIN, MMP-9, and VEGF (Figure 5). To clarify the pathway involved, we used the AMPK inhibitor dorsomorphin, and showed that inhibiting AMPK activity enhanced the secretion of EMMPRIN, MMP-9, and VEGF. Moreover, the combination of the AMPK inhibitor and metformin was not different from the effects of the AMPK inhibitor alone (Figure 5), suggesting that metformin works upstream of AMPK, probably on LKB1, while the AMPK inhibitor directly affects the AMPK enzyme. Thus, we suggest that in normal glycemic conditions, AMPK inhibits EMMPRIN, and consequently also VEGF and MMP-9, keeping angiogenesis in check, whereas in hyperglycemic conditions, AMPK is inhibited and the secretion of the pro-angiogenic factors is enhanced. AMPK has been previously suggested to regulate EMMPRIN via the activation of MAPKs [58,59], but not in the context of hyperglycemia. Other studies demonstrated that high levels of glucose and AGEs enhanced EMMPRIN expression and glycosylation in THP-1 monocytic cells and in adipocytes, via RAGE and the NF-κB pathway, but not via AMPK [60,61]. Thus, the crosstalk between the different pathways is very complex, and probably differs between cell types and environmental conditions. In EaHy926 cells, metformin reduced EMMPRIN and MMP-9 secretion even in low glucose conditions, suggesting that AMPK activity may be additionally regulated and fine-tuned by other kinases, and reflecting the differences between the two endothelial cells. Importantly, in both endothelial cell lines, metformin reverses the effect of high glucose and restores the secretion of the pro-angiogenic factors to normal levels. This might suggest that metformin may be used to improve the pathological angiogenesis observed in retinopathy, for example. However, the impact of systemic metformin on other tissues that exhibited reduced angiogenesis must also be explored. 

Importantly, dorsomorphin is not a specific inhibitor of AMPK, and can inhibit other kinases, especially kinases that are activated in the BMP-signaling pathway. Hence, it cannot be used as a single agent to study AMPK activity [62], and metformin, a known AMPK activator, was also used. However, in view of the complex crosstalk between different pathways, we cannot rule out the possibility that kinases other than AMPK, such as activin-like receptor kinases (ALKs), are inhibited by dorsomorphin and contribute to the overall inhibitory effect. Additionally, our findings are limited to the in vitro setting that uses only two types of cell lines, endothelial and monocytic, in co-culture. These findings await further investigation in an animal model in vivo, which was out of the scope of the current study.

## 5. Conclusions

We show that macrovascular and microvascular endothelial cells mostly respond similarly to high levels of glucose and the presence of monocytic cells by increasing their secretion of the pro-angiogenic factors EMMPRIN, VEGF, and MMP-9. We implicate the reduced activity of AMPK in the increased EMMPRIN secretion that leads to the increased secretion of VEGF and MMP-9 and enhanced angiogenic potential. However, the angiogenic paradox cannot be explained by the minor differences observed between the macrovascular and microvascular endothelial cells, and we speculate that tissue-specific factors, such as the interactions with different cell types, the microenvironment milieu in the tissue, or the interaction with other ECM proteins might play a role. 

## Data Availability

The data presented in this study are available in the article.

## Supplementary Materials

The following supporting information can be downloaded at: https://www.mdpi.com/article/10.3390/biomedicines12040706/s1, Figure S1: The anti-EMMPRIN antibody (h161-pAb) reduces the secretion of VEGF and MMP-9 from the three mono-cultured cells. The human endothelial cells EaHy926, HMEC-1, or the monocytic U937 cell line (20,000 cells/well) were incubated in the indicated glucose concentrations with or without h161-pAb (2 ng/mL) for 48 h, and the concentrations of (A–C) MMP-9 or (D–F) VEGF were measured in the supernatants. The means ± SEM are presented (*n* = 4–5). Data were analyzed using the one-way ANOVA followed by Bonferroni’s post hoc test. Figure S2: The anti-EMMPRIN antibody (h161-pAb) decreases HMEC-1 angiogenic potential. HMEC-1 endothelial cells (20,000/well) were seeded and allowed to grow to confluency overnight. Then, a scratch was made with a toothpick, and non-adherent cells were washed away. The cells were then incubated for additional 18 h in full medium and with supernatants (diluted 2.5:1) derived from HMEC-1 cells that were previously incubated alone or in co-culture with glucose and with or without the h161-pAb (2 ng/mL). Representative images at (A) a concentration of 5.5 mmole/L glucose or (B) 25 mmole/L glucose, and (C) quantitative analysis of the assays (*n* = 4–5). The migration distance was calculated as before. The means ± SEM are presented, and data were analyzed using the one-way ANOVA followed by Bonferroni’s post hoc test. Table S1: Detection range, the intra- and inter-assay coefficient of variations (CVs) for the ELISA kits used.
