# Supplementary material for: Glucose Promotes EMMPRIN/CD147 and the Secretion of Pro-Angiogenic Factors in a Co-Culture System of Endothelial Cells and Monocytes
Source: Biomedicines. 2024 Mar 22;12(4):706. doi: 10.3390/biomedicines12040706 (PMC11047830; doi:10.3390/biomedicines12040706)
Supplement: Supplementary file 1 [file biomedicines-12-00706-s001.zip › biomedicines-2895119-supplementary.pdf]

Glucose promotes EMMPRIN/CD147 and the secretion of pro-angiogenic factors in a co-culture system of endothelial cells and monocytes

Supplementary Materials

Table S1: detection range, the intra- and inter-assay coefficient of variations (CVs) for the ELISA kits used

Kit	pAMPK	EMMPRIN	VEGF	MMP-9
Assay range (pg/ml)	62.5-4,000	62.5-4,000	31.2-2,000	31.2-2,000
Inter-assay CV (%)	9.612%	11.09%	11.33%	12.53%
Intra-assay CV (%)		10.97%	9.983%	6.764%

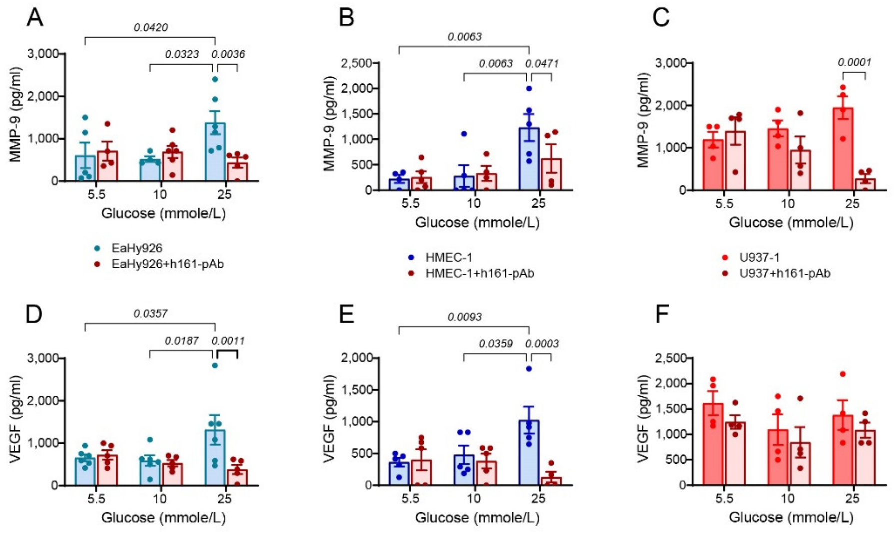

Figure S1: The anti-EMMPRIN antibody (h161-pAb) reduces the secretion of VEGF and MMP-9 from the three mono-cultured cells. The human endothelial cells EaHy926, HMEC-1 or the monocytic U937 cell line (20,000 cells/well) were incubated in the indicated glucose concentrations with or without h161-pAb (2 ng/ml) for 48 hours, and the concentrations of (**A-C**) MMP-9 or (**D-F**) VEGF were measured in the supernatants**.** The means ± SEM are presented (n=4-5). Data were analyzed using the one-way ANOVA followed by Bonferroni's post-hoc test.

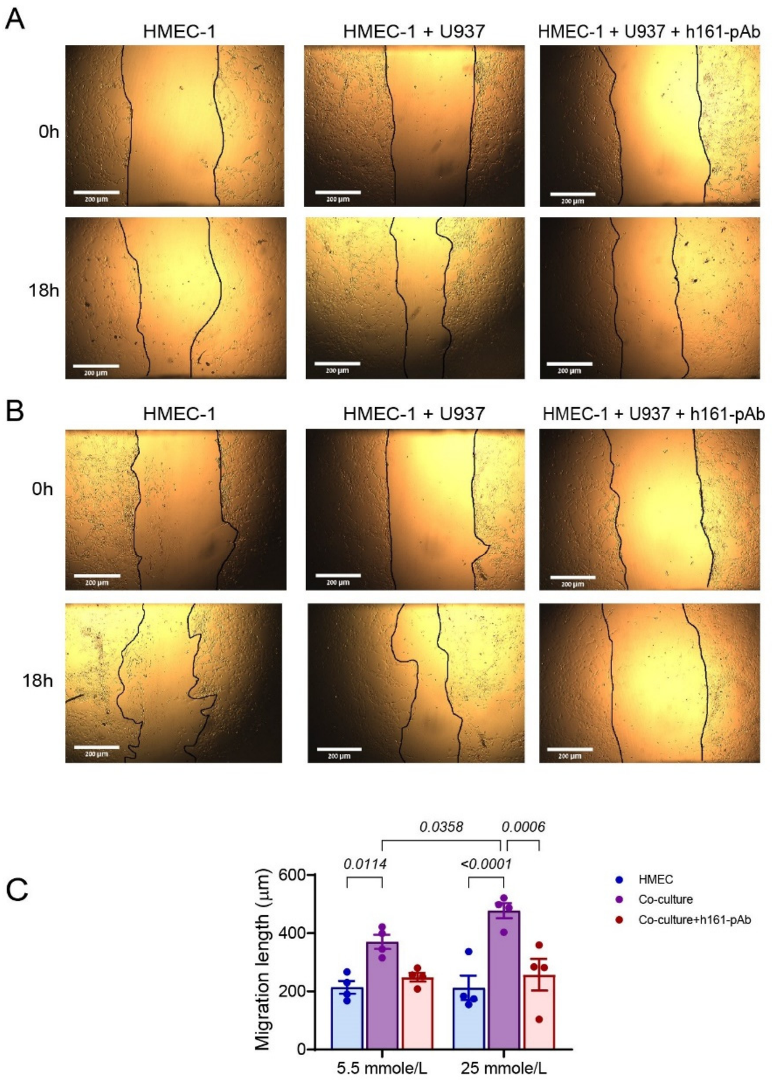

Figure S2: The anti-EMMPRIN antibody (h161-pAb) decreases HMEC-1 angiogenic potential. HMEC-1 endothelial cells (20,000/well) were seeded and allowed to grow to confluency overnight. Then, a scratch was made with a toothpick, and non-adherent cells were washed away. The cells were then incubated for additional 18h in full medium and with supernatants (diluted 2.5:1) derived from HMEC-1 cells that were previously incubated alone or in co-culture with glucose and with or without the h161-pAb (2 ng/ml). Representative images at (**A**) a concentration of 5.5 mmole/L glucose or (**B**) 25 mmole/L glucose, and (**C**) quantitative analysis of the assays (n=4-5). The migration distance was calculated as before. The means ± SEM are presented, and data were analyzed using the one-way ANOVA followed by Bonferroni's post-hoc test.
